# Esomeprazole inhibits EGF-induced invasion and migration of gastric cancer cells

**DOI:** 10.7717/peerj.21675

**Published:** 2026-09-11

**Authors:** Fangfang Huang, Jinfeng Du, Xuanruo Zhang, Mengru Tian, Qiangqiang Lv, Xiufeng Chu, Hongqiao Zhang, Zisen Zhang

**Affiliations:** 1 Zhengzhou Key Laboratory of Lipid Metabolism and Precision Diagnosis & Treatment in Oncology, Oncology Department, the Fifth Affiliated Hospital of Zhengzhou University, Zhengzhou, Henan, China; 2 State Key Laboratory of Metabolic Dysregulation & Prevention and Treatment of Esophageal Cancer, Tianjian Laboratory of Advanced Biomedical Sciences, Zhengzhou, Henan, China; 3 Chongqing Emergency Medical Center, Chongqing, China; 4 Henan Key Laboratory of Health Medicine and Liver Disease Prevention & Treatment, Henan Institute of Medical and Pharmaceutical Sciences, Zhengzhou University, Zhengzhou, Henan, China

**Keywords:** Gastric cancer, Esomeprazole, Epidermal growth factor, Invasion, Migration, Afatinib, EGFR/AKT/mTOR

## Abstract

**Background:**

Gastric cancer (GC) is one of the most prevalent and lethal malignancies worldwide, with invasion and metastasis being the key factors contributing to its poor prognosis and high mortality rates. Epidermal growth factor (EGF)-induced activation of EGFR and downstream AKT/mTOR signaling plays a central role in promoting cancer cell invasion and migration. While epidermal growth factor receptor (EGFR)-targeted therapies such as afatinib effectively inhibit these processes, the potential of esomeprazole (ESO) to modulate GC metastasis has not been fully elucidated. This study aims to investigate the inhibitory effect of ESO on EGF-induced invasion and migration of gastric cancer cells, and its relationship with the EGFR/protein kinase B (AKT)/mechanistic target of rapamycin (mTOR) signaling pathway.

**Methods:**

Cell viability, invasion, migration and protein expression were assessed using cell counting kit-8 (CCK-8) assay, Transwell assay, wound healing assay and western blot.

**Results:**

ESO significantly reduced migration and invasion in GC cells, as well as the expression levels of N-cadherin, MMP2, MMP9, Vimentin, p-EGFR, p-AKT, and p-mTOR induced by EGF. The combination of ESO and afatinib (an irreversible EGFR inhibitor) led to a more pronounced reduction in cell viability, invasion, migration, and protein expression compared to either treatment alone.

**Conclusions:**

ESO inhibits EGF-induced invasion and migration in GC cells potentially *via* the EGFR/AKT/mTOR pathway, offering a potential new strategy for GC treatment.

## Introduction

Gastric cancer (GC) is a major global health burden, with over 968,000 new cases and nearly 660,000 deaths in 2022, ranking fifth in terms of both incidence and mortality worldwide (Bray et al., 2024). The development of GC is influenced by a complex interplay of risk factors, including *Helicobacter pylori* infection, lifestyle factors such as smoking and high-salt diet, genetic predisposition, and aging (Halmai & Carvajal-Carmona, 2023). Although the medical care nowadays has been far developed, most GC patients are diagnosed at advanced stages with poor prognosis (Guan, He & Xu, 2023). The current therapeutic options for advanced GC, including systemic chemotherapy, targeted therapy, and immunotherapy, can be limited by drug resistance, severe side effects, tumor heterogeneity, and metastasis (Sundar et al., 2025). These challenges highlight the need to explore novel therapeutic agents, targets, and combination strategies that can effectively suppress tumor progression, particularly the lethal processes of invasion and metastasis.

The invasive and migratory abilities of GC cells are key determinants of tumor progression, leading to local infiltration, lymph node involvement, and distant organ dissemination, which contribute to its poor prognosis and overall survival (Ara et al., 2016; Haoyuan & Yanshu, 2020).

GC cell invasion and migration are highly dynamic and multistep processes governed by a complex interplay of molecular and cellular events. These processes are closely associated with epithelial-mesenchymal transition (EMT), extracellular matrix (ECM) remodeling, and cytoskeletal reorganization. In particular, the upregulation of key mesenchymal and proteolytic markers, including N-cadherin, vimentin, matrix metalloproteinase-2 (MMP-2), and MMP-9, has been consistently linked to enhanced invasive and metastatic potential in GC (Meng, Hu & Xu, 2023). These molecules facilitate tumor cell detachment, degradation of the surrounding ECM, and subsequent dissemination to distant sites. Therefore, elucidating the molecular mechanisms underlying GC cell invasion and migration is of critical importance for the development of effective therapeutic strategies and the improvement of patient outcomes.

Recent studies have shown that the overexpression of Epidermal growth factor (EGF) and epidermal growth factor receptor (EGFR) is associated with increased invasion and metastasis in GC cells, as well as shorter survival time and higher recurrence risks (Lei et al., 2022; Li, Zheng & Xue, 2019). EGF, the natural ligand for EGFR, can drive tumor cell invasion and migration by continuously activating EGFR (Cabezas-Sáinz et al., 2020). Although specific EGFR inhibitors, such as afatinib, have been developed, their efficacy as monotherapy in GC is highly limited due to compensatory survival mechanisms and drug resistance, underscoring the necessity for innovative combination therapies (Ebert et al., 2019).

Hyperactivation of EGFR upregulates several downstream pathways including Ras/Raf/MAPK pathway and AKT/mTOR pathway (Zhang et al., 2023). Notably, the AKT/mTOR pathway plays an important regulatory role in tumor invasion and migration (Zhang et al., 2022). Specifically, upon EGFR activation, AKT is recruited to the plasma membrane and phosphorylated, and then directly phosphorylates mTOR, thereby initiating the AKT/mTOR pathway (Liu et al., 2021; Zheng et al., 2014). Subsequently, this pathway drives cell invasion and migration by inducing EMT and upregulating MMPs to degrade the extracellular matrix (Hu et al., 2024; Longobardi et al., 2026; Yang et al., 2024; Zhang et al., 2025).

Esomeprazole (ESO), a proton pump inhibitor traditionally used for acid-suppressive therapy, has recently emerged as a promising anti-tumor agent (Wang et al., 2015). The proposed anticancer effects are primarily attributed to its ability to neutralize the acidic tumor microenvironment by inhibiting V-ATPase in tumor cells, or to reverse chemoresistance and impair tumor survival (Joha et al., 2025; Matsumura et al., 2022; Wang et al., 2015). In parallel, our previous study shows that ESO can inhibit the growth of GC cells by downregulating EGFR (Du et al., 2022). In this study, we aimed to investigate whether ESO reversed the EGF-induced GC cell invasion and migration, and whether the inhibitory effect correlated with the inactivation of EGFR/AKT/mTOR pathway and downregulation of key effector proteins involved in metastasis. Furthermore, we explored the synergistic effects of ESO combined with afatinib in inhibiting GC cell invasion and migration, laying the groundwork for future studies on the potential of this combination therapy for treating advanced GC or overcoming afatinib resistance.

## Materials and Methods

### Reagents and chemicals

ESO was obtained from AstraZeneca Pharmaceutical Co., Ltd. (Changzhou, China). Afatinib (S1011) was purchased from Selleck Chemicals (Houston, TX, USA). Recombinant human Epidermal Growth Factor (EGF, AF-100-15) was sourced from PeproTech (Cranbury, NJ, USA). Cell Counting Kit-8 (CCK-8, BS350B) and RIPA lysis buffer (BL504A) were acquired from Biosharp Life Sciences (Hefei, China). A 3D cell culture matrix kit (B-P-00002-2) was purchased from Biozellen Scientific (TX, USA). Protease and phosphatase inhibitor cocktail (P002) was purchased from NCM Biotech (Jiangsu, China). Enhanced Chemiluminescence (ECL) substrate (P0018FS) was purchased from Beyotime Biotechnology (Shanghai, China).

Primary antibodies used for Western blot analysis included: anti-N-cadherin (1:1000, AF4039; Affinity Biosciences, Cincinnati, OH, USA), anti-Vimentin (1:1000, T55134; Abmart, Shanghai, China), anti-MMP2 (1:1000, T57164; Abmart, Shanghai, China), anti-MMP9 (1:1000, T55205; Abmart, Shanghai, China), anti-Phospho-EGFR (Tyr1068, 1:1000, 18986-1-AP-50; Proteintech, Wuhan, China), anti-Phospho-AKT (Ser473, 1:1000, AF0016; Affinity Biosciences, Cincinnati, OH, USA), anti-Phospho-mTOR (Ser2448, 1:1000, ab09268-40; Abcam, Cambridge, UK), and anti-GAPDH (1:4000, 10494-1-AP; Proteintech, Wuhan, China). Horseradish peroxidase (HRP)-conjugated goat anti-rabbit IgG (H + L) secondary antibody (1:5000, SA0001; Proteintech, Wuhan, China) was used as the secondary antibody.

### Cell culture

Human GC cell lines AGS and HGC-27 were obtained from the Shanghai Cell Bank of the Chinese Academy of Sciences (Shanghai, China). Cells were cultured in RPMI-1640 medium (HGC-27) or F-12K medium (AGS) supplemented with 10% fetal bovine serum (FBS) and 1% penicillin-streptomycin at 37 °C in a humidified atmosphere with 5% CO_2_.

### Cell viability assay

Cell viability was assessed using the CCK-8 assay. Briefly, AGS and HGC-27 cells were seeded in 96-well plates at a density of 4–6 × 10^3^ cells/well and treated with indicated agents for specified time periods. After treatment, 10 µL of CCK-8 reagent was added to each well and incubated for 1–4 h. The absorbance was measured at 450 nm using a microplate reader.

### Wound healing assay

Cells were seeded in 6-well plates and grown to 90–100% confluence. A sterile pipette tip was used to create a scratch wound. After washing with PBS, cells were treated with ESO, EGF, afatinib, or their combinations. Images were captured at 0 and 24 h using an inverted microscope. The migration rate was calculated as:

$\rm {Migration \, rate \, (\%) = [(A0 - A24) / A0] \times 100}$

where A0 and A24 represent the wound area at 0 and 24 h respectively.

### Transwell migration and invasion assays

For migration assays, 2 × 10^4^ cells in serum-free medium were seeded into the upper chamber of a Transwell insert. For invasion assays, the upper chamber was pre-coated with Matrigel. The lower chamber contained medium with 10% FBS as a chemoattractant. After 24 h of incubation, cells that migrated to or invaded the lower surface were fixed with 4% paraformaldehyde, stained with crystal violet, and counted under a microscope in five random fields.

### Western blot analysis

Cells were lysed in RIPA buffer containing protease and phosphatase inhibitors. Protein concentrations were determined using a BCA assay. Equal amounts of protein were separated by SDS-PAGE and transferred to PVDF membranes. After blocking with 5% non-fat milk, membranes were incubated with primary antibodies overnight at 4 °C, followed by HRP-conjugated secondary antibodies. Protein bands were visualized using an ECL detection system and quantified with ImageJ software.

### Statistical analysis

All experiments were performed in at least three independent replicates. Data were presented as mean ± SD. Statistical analyses were performed using SPSS 21.0 and GraphPad Prism 8.0. Differences between two groups were analyzed by Student’s t-test, and multiple comparisons were performed using one-way ANOVA with Bonferroni *post-hoc* test. A *P*-value < 0.05 was considered statistically significant.

## Results

### ESO inhibits cell viability, migration and invasion of GC cells

**Figure 1:**
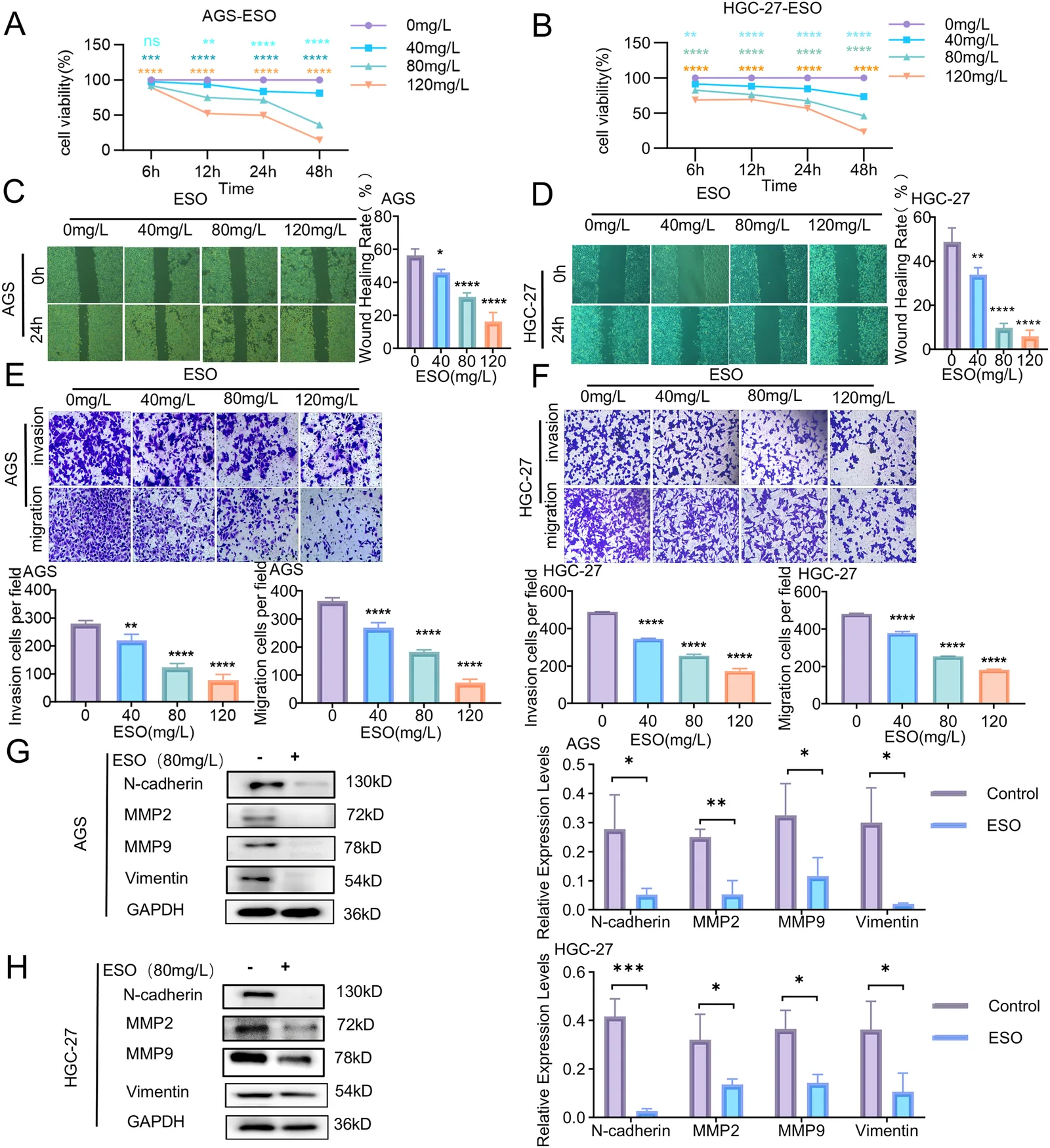
ESO inhibits cell viability, migration and invasion of AGS and HGC-27 cells. (A, B) CCK-8 assay showed the viability of AGS and HGC-27 cells treated with different concentrations (0, 40, 80, and 120 mg/L) of ESO for 6, 12, 24, and 48 h. (C, D) Wound healing assays demonstrated the migration of AGS and HGC-27 cells after 24 h of ESO treatment (magnification 100×). (E, F) Transwell migration and invasion assays were conducted to detect the migration and invasion of AGS and HGC-27 cells treated with ESO for 24 h (magnification 100×). (G, H) The protein expression levels of N-cadherin, MMP2, MMP9 and Vimentin were detected by Western blot after 24 h of ESO treatment in AGS cells (G) and HGC-27 cells (H). Statistical significance: *P < 0.05, **P < 0.01, ***P < 0.001, ****P < 0.0001.

To examine the effects of ESO on GC cell viability, CCK-8 assays were performed on AGS and HGC-27 cells treated with different concentrations of ESO (0, 40, 80, and 120 mg/L) at 6, 12, 24, and 48 hours (h). The results demonstrated that ESO significantly reduced the viability of both cell lines in a dose- and time-dependent manner (Figs. 1A, 1B), indicating its inhibitory effect on cell proliferation. To further assess the impact of ESO on cell motility, we performed wound healing assays, Transwell invasion and migration assays after ESO treatment for 24 h. Our results showed that ESO effectively inhibited both cell migration and invasion in AGS and HGC-27 cells in a dose-dependent manner (Figs. 1C–1F), suggesting that ESO effectively impaired GC cell motility. Then, we analyzed the expression of key migration- and invasion-related proteins in AGS and HGC-27 cells by Western blot to explore the underlying mechanism. The results revealed that the protein expression levels of N-cadherin, MMP2, MMP9, and Vimentin were significantly decreased after treatment with ESO (80 mg/L) for 24 h in both AGS and HGC-27 cells (Figs. 1G, 1H). These findings demonstrated that ESO exerted multifaceted inhibitory effects on GC cells by suppressing cell viability, migration, and invasion.

### EGF promotes migration and invasion of GC cells independent of proliferative effect

**Figure 2:**
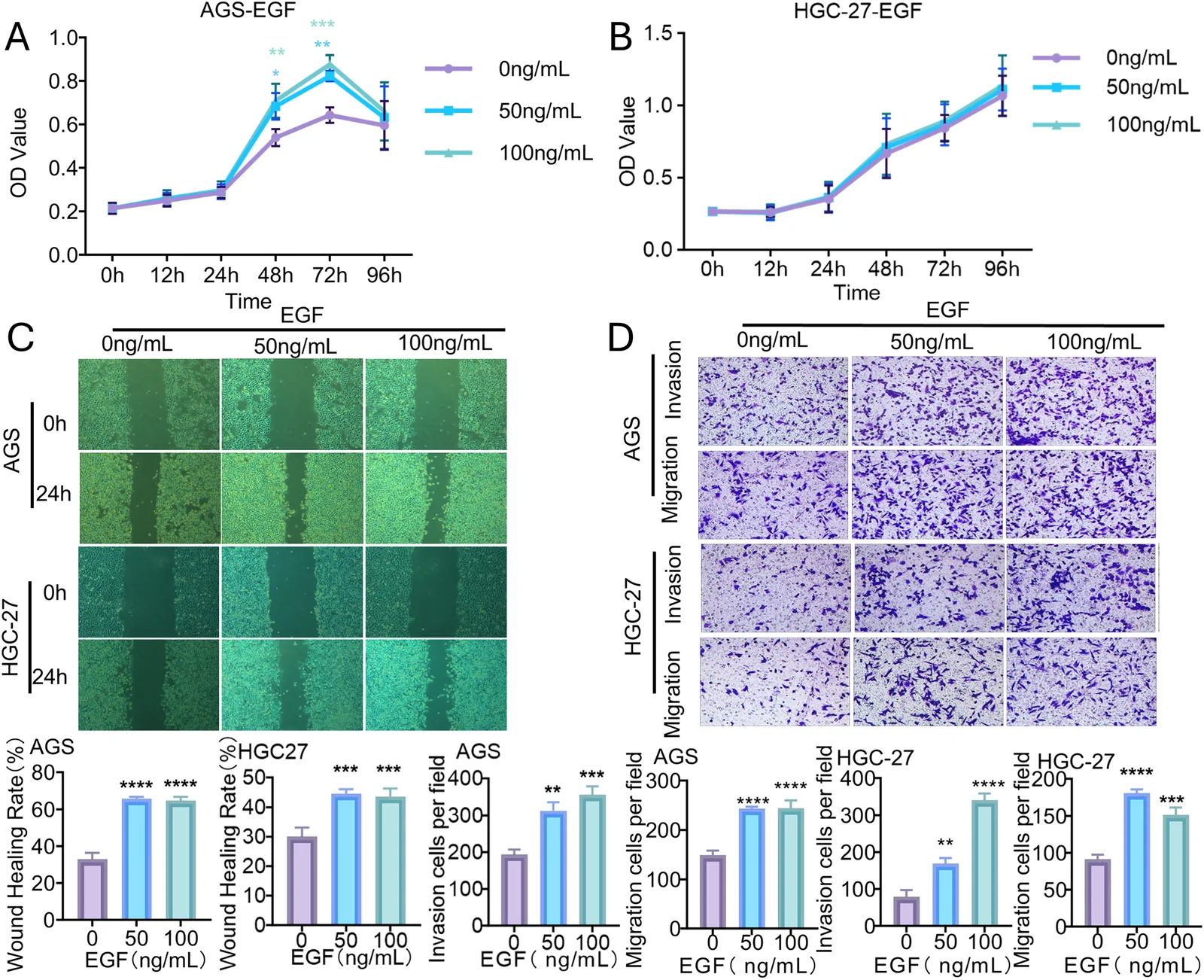
EGF promotes migration and invasion of GC cells independent of proliferative effect. (A, B) The viability of AGS and HGC-27 cells was measured using the CCK-8 assay after treatment with increasing concentrations of EGF (0, 50, and 100 ng/mL) for 0, 12, 24, 48, 72, and 96 h. (C) Wound healing assay showed the migratory capacity of AGS and HGC-27 cells after EGF treatment for 24 h (magnification 100×). (D) Transwell assay assessed the number of migration cells and invasion cells in AGS and HGC-27 cells after exposure to EGF for 24 h (magnification 100×). Statistical significance: *P < 0.05, **P < 0.01, ***P < 0.001, ****P < 0.0001.

Next, we investigated whether EGF could induce migration and invasion of GC cells by performing CCK-8 assays, wound healing assays, and Transwell assays. Cells were treated with different concentrations of EGF (0, 50, and 100 ng/mL), and cell viability was assessed at multiple time points (0, 12, 24, 48, 72, and 96 h) using the CCK-8 assay. In AGS cells, EGF significantly increased cell viability at 48 and 72 h, while no significant changes were observed at 12, 24, or 96 h (Fig. 2A). In contrast, HGC-27 cell viability remained unaffected by EGF treatment at all tested time points (Fig. 2B). The wound healing assay demonstrated that EGF enhanced scratch healing rate in AGS and HGC-27 cells at 24 h, although this effect did not exhibit clear dose dependence (Fig. 2C). To further evaluate cell motility, Transwell migration and invasion assays were conducted. EGF-treated cells displayed significantly increased numbers of migrating and invading cells after 24 h, again without an evident dose-dependent pattern (Fig. 2D). The results suggested that EGF significantly enhanced the migratory and invasive capabilities of GC cells, whereas cell proliferation did not contribute to this process.

### ESO inhibits migration and invasion induced by EGF in GC cells

**Figure 3:**
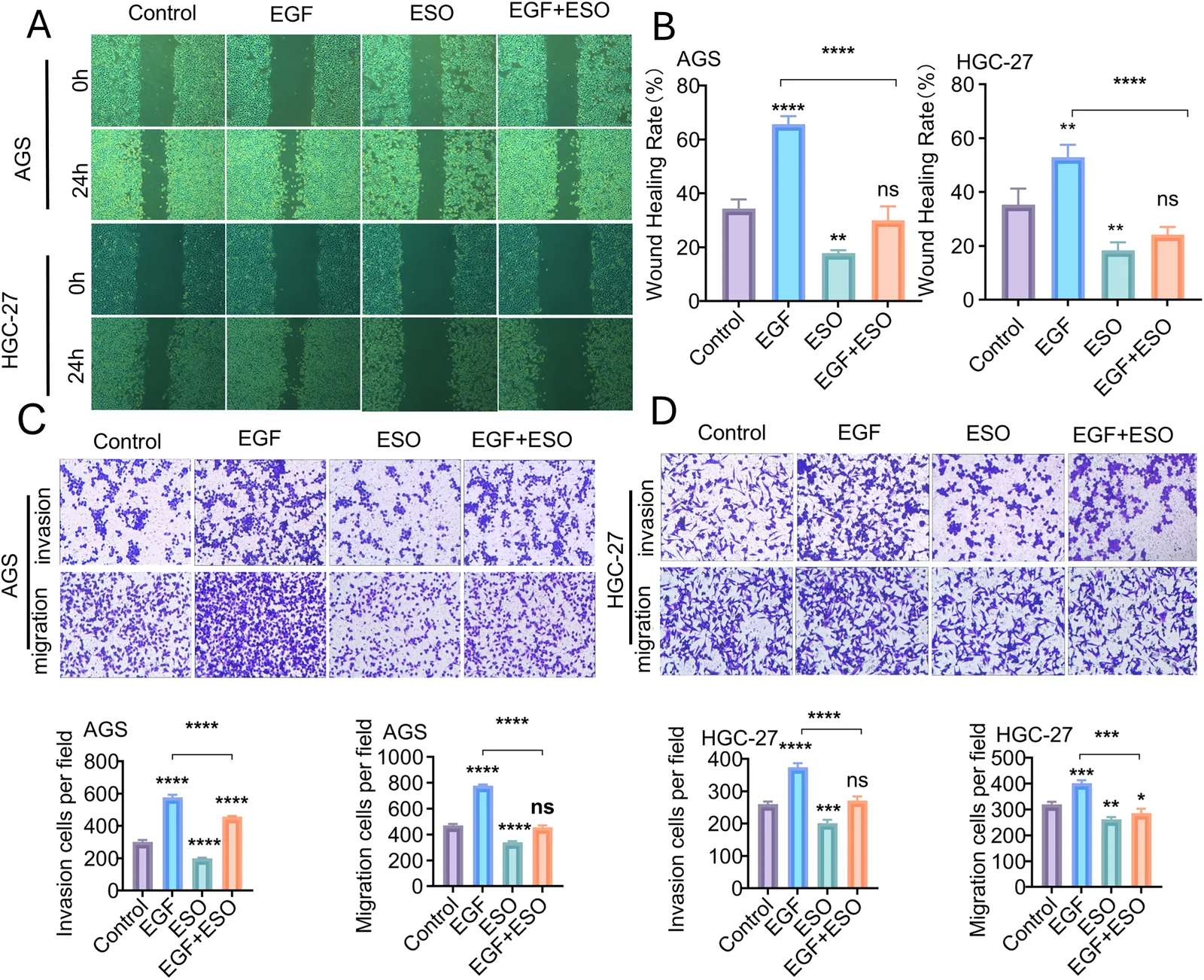
ESO inhibits migration and invasion induced by EGF in GC cells. (A, B) Scratch assay was used to detect the migratory capacity of AGS and HGC-27 cells respectively treated with EGF (50 ng/mL), ESO (80 mg/L), EGF combined with ESO for 24 h (magnification 100×). (C, D) Transwell assay showed the number of migration and invasion cells in AGS and HGC-27 cells after exposure to EGF, ESO, EGF combined with ESO for 24 h (magnification 100×). Statistical significance: *P < 0.05, **P < 0.01, ***P < 0.001, ****P < 0.0001.

Then, wound healing assay confirmed that ESO significantly suppressed wound healing rate enhanced by EGF in AGS and HGC-27 cells (Figs. 3A, 3B). Transwell migration and invasion assays revealed that ESO treatment markedly reduced the EGF-induced increase in migrating and invading cell numbers in AGS and HGC-27 cells (Figs. 3C, 3D). These findings collectively indicated that ESO effectively antagonized the enhancement of migratory and invasive capabilities in GC cells promoted by EGF.

### ESO inhibits the EGF-induced expression of migration- and invasion-related proteins and EGFR/AKT/mTOR pathway

**Figure 4:**
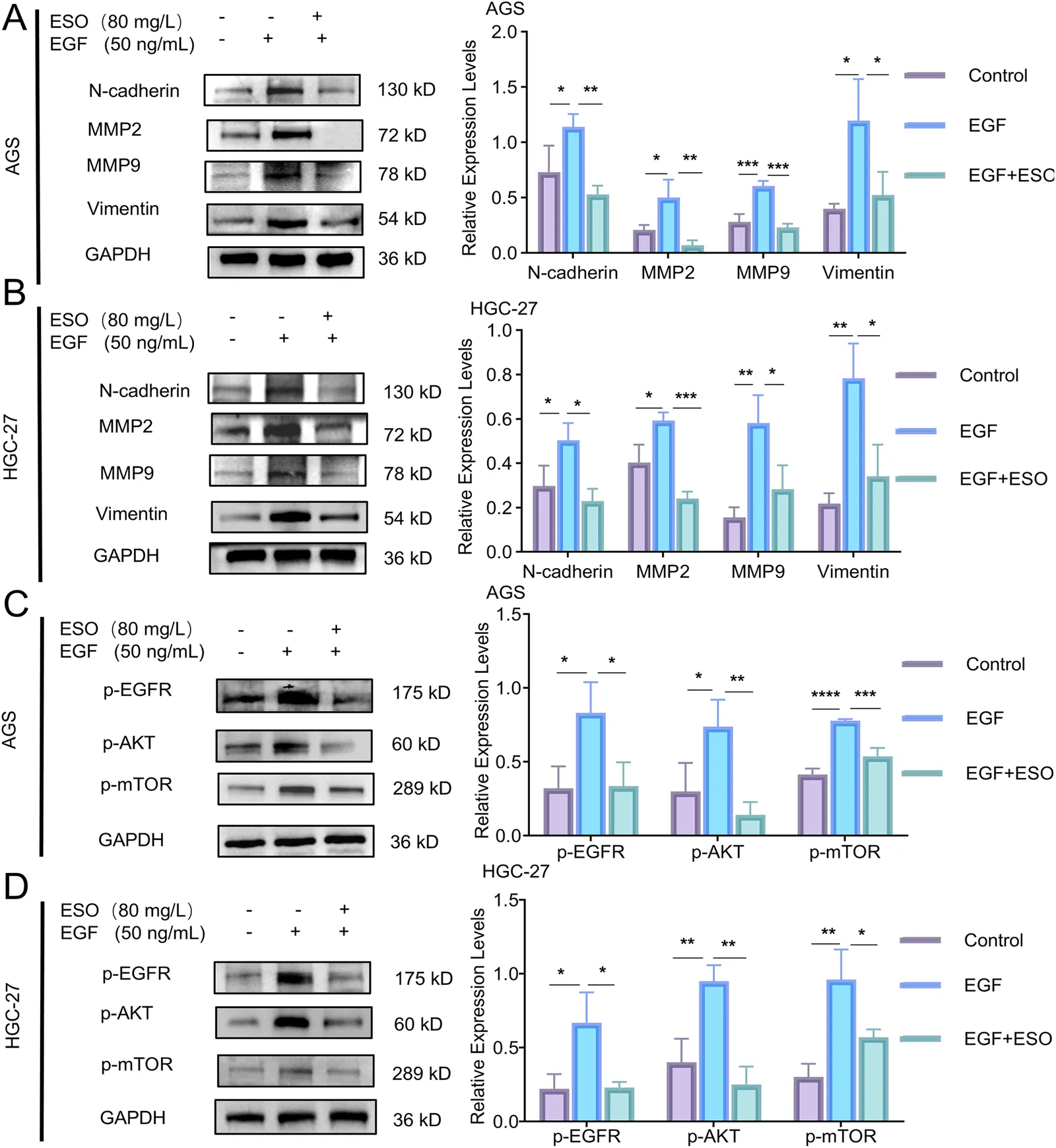
ESO inhibits the EGF-induced expression of migration- and invasion-related proteins and EGFR/AKT/mTOR pathway. (A, B) Western blot was performed to measure relative protein expression levels of N-cadherin, MMP2, MMP9, Vimentin in AGS (A) and HGC-27 (B) cells after treatment with EGF (50 ng/mL), ESO (80 mg/L) and EGF combined with ESO for 24 h. (C, D) Western blot showed relative protein expression levels of p-EGFR, p-AKT and p-mTOR in AGS (C) and HGC-27 (D) cells after exposure to EGF, ESO and EGF combined with ESO (80 mg/L) for 24 h. Statistical significance: *P < 0.05, **P < 0.01, ***P < 0.001, ****P < 0.0001.

To elucidate the potential mechanism, we performed Western blot to measure the protein expression levels of migration- and invasion-related proteins (N-cadherin, MMP2, MMP9 and Vimentin) and key phosphorylated proteins in the EGFR/AKT/mTOR pathway (p-EGFR, p-AKT, p-mTOR) in AGS and HGC-27 cells. Western blot showed that EGF exposure pronouncedly upregulated the expression levels of N-cadherin, MMP2, MMP9, Vimentin (Figs. 4A, 4B), as well as p-EGFR, p-AKT, and p-mTOR (Figs. 4C, 4D) in AGS and HGC-27 cells. In contrast, ESO treatment reversed these changes, considerably reducing N-cadherin, MMP2, MMP9, Vimentin (Figs. 4A, 4B), p-EGFR, p-AKT, p-mTOR (Figs. 4C, 4D) expression. These findings suggested that ESO suppressed EGF-induced migration and invasion in association with inactivation of EGFR/AKT/mTOR pathway and downregulation of migration-related and invasion-related proteins.

### ESO combined with afatinib enhances the suppression of invasion and migration of GC cells compared to monotherapy

**Figure 5:**
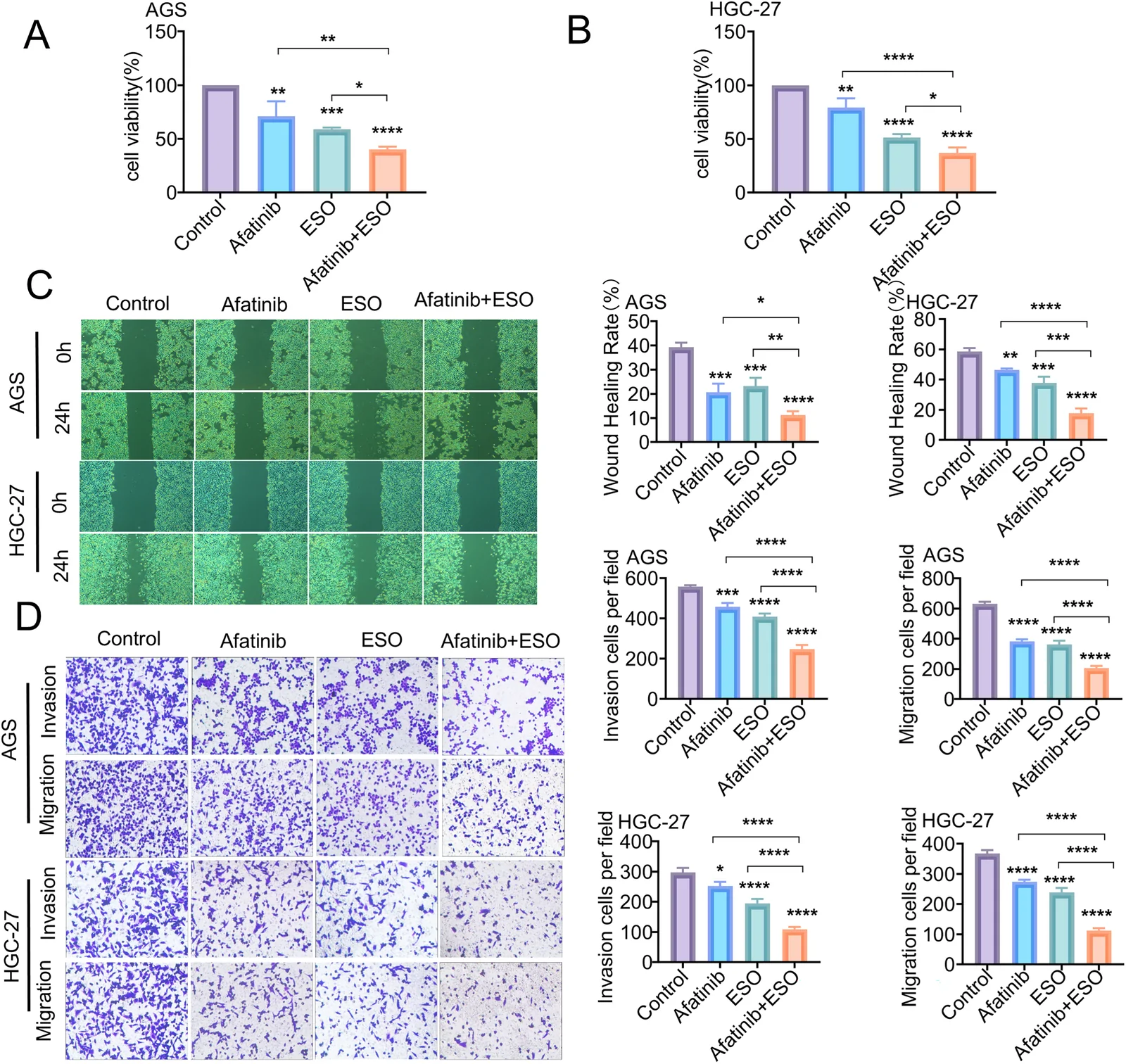
ESO combined with afatinib enhances the suppression of invasion and migration of GC cells compared to monotherapy. (A, B) CCK-8 assay evaluated the viability of AGS and HGC-27 cells respectively treated with afatinib (2 μM), ESO (80 mg/L) and afatinib combined ESO for 24 h. (C) Scratch assay showed the migration of AGS and HGC-27 cells treated with afatinib, ESO and afatinib combined with ESO for 24 h (magnification 100×). (D) Transwell assays were performed to detect the migration and invasion of AGS and HGC-27 cells treated with afatinib, ESO and their combination for 24 h (magnification 100×). Statistical significance: *P < 0.05, **P < 0.01, ***P < 0.001, ****P < 0.0001.

To further confirm whether targeting EGFR can potentiate the antitumor effects of ESO, we evaluated the impact of combining the clinical EGFR inhibitor afatinib with ESO on the viability, migration, and invasion of AGS and HGC-27 cells using CCK-8, wound healing, and Transwell assays. The CCK-8 assay showed that the combination of ESO and afatinib resulted in a more significant suppression of cell viability compared to treatment with either agent alone in both AGS and HGC-27 cells (Figs. 5A, 5B). Consistently, wound healing and Transwell assays confirmed that the combination treatment exerted a stronger inhibitory effect on cell migration and invasion than monotherapy (Figs. 5C, 5D). These results corroborated that the combined application of ESO and afatinib produced an enhanced inhibitory effect on cell growth, invasion and migration compared to afatinib or ESO alone.

### ESO and afatinib potentiate suppression of migration- and invasion-related proteins and the EGFR/AKT/mTOR pathway

**Figure 6:**
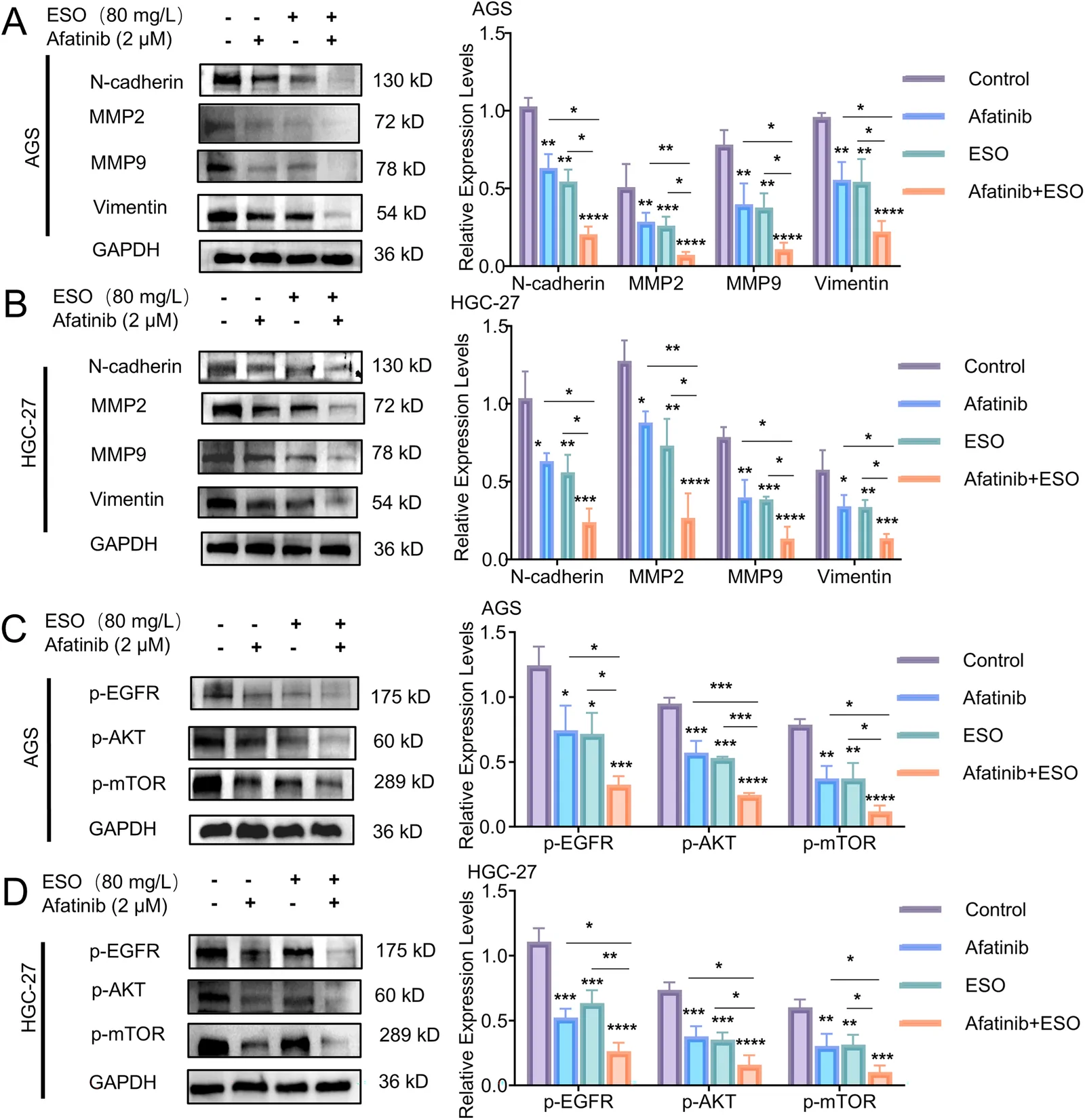
ESO and afatinib potentiate suppression of migration- and invasion-related proteins and the EGFR/AKT/mTOR pathway. (A, B) Western blot was performed to measure relative protein expression levels of N-cadherin, MMP2, MMP9, Vimentin in AGS (A) and HGC-27 (B) cells after treatment with afatinib (2 μM), ESO (80 mg/L) and afatinib combined ESO for 24 h. (C, D) Western blot showed relative protein expression levels of p-EGFR, p-AKT and p-mTOR in AGS (C) and HGC-27 (D) cells after exposure to afatinib (2 μM), ESO (80 mg/L) and afatinib combined ESO for 24 h. Statistical significance: ns, not significant; *P < 0.05, **P < 0.01, ***P < 0.001, ****P < 0.0001.

Subsequently, we assessed the expression of migration- and invasion-related proteins (N-cadherin, MMP2, MMP9, Vimentin) and phosphorylated proteins (p-EGFR, p-AKT, p-mTOR) in the EGFR/AKT/mTOR signaling pathway. Western blot analysis showed that, compared to treatment with afatinib or ESO alone, the combination therapy resulted in a more pronounced reduction in the expression levels of N-cadherin, MMP2, MMP9, Vimentin (Figs. 6A, 6B), as well as p-EGFR, p-AKT, and p-mTOR (Figs. 6C, 6D) in AGS and HGC-27 cells. These findings indicated that the enhanced anticancer efficacy of ESO and afatinib combination was associated with inhibition of the EGFR/AKT/mTOR signaling pathway and its downstream effectors involved in migration and invasion.

## Discussion

GC is one of the most common and lethal malignancies worldwide, ranking fifth in both incidence and mortality (Bray et al., 2024). Among the factors contributing to cancer progression, invasion and migration are recognized as the critical challenges in GC (Aghiorghiesei et al., 2025). These processes are associated with the development of local infiltration, lymphatic and hematogenous dissemination, and ultimately distant metastasis (Sun et al., 2019). Notably, distant metastasis is the primary cause of treatment failure and death in GC patients (He et al., 2014). Therefore, a deeper understanding of the mechanisms regulating invasion and migration is essential for identifying novel therapeutic targets and improving clinical outcomes in GC.

Our study showed that ESO treatment suppressed migration and invasion of GC cells in a dose-dependent manner. ESO also demonstrated its ability to counteract EGF-induced motility of GC cells. This aligns with prior reports that proton pump inhibitors (PPIs) like ESO exert antitumor effects. For instance, ESO potentiates cisplatin-induced cytotoxicity in SNU-1 GC cells (Joha et al., 2025). It also reverses multidrug resistance in SGC7901/MDR GC cells and enhances chemosensitivity to antitumor drugs (Chen et al., 2018). Beyond GC, ESO alone exhibits remarkable therapeutic efficacy in hormone-sensitive breast tumors and further modulates the tumor immune microenvironment by increasing CD8^+^ T lymphocyte infiltration while decreasing M2 macrophages (Balza et al., 2022). ESO has also been shown to inhibit liver inflammation and carcinogenesis by suppressing lipopolysaccharide-induced NF-κB and farnesoid X receptor (Lu et al., 2025). Additionally, in head and neck squamous cell carcinoma, ESO potentiates the cytotoxic effects of ionizing radiation, thereby improving tumor control in preclinical models (Hebert et al., 2021). These findings suggest that ESO may inhibit cancer progression through multiple mechanisms, and our study emphasizes its relevance to metastasis-associated pathways.

Our data revealed that ESO downregulated key proteins associated with EMT and ECM remodeling, including N-cadherin, vimentin, MMP-2, and MMP-9. These proteins are well-established drivers of metastatic potential in GC, and their upregulation correlates with poor prognosis and aggressive tumor behavior (Guo et al., 2021). For example, MMP-2 and MMP-9 overexpression facilitates GC cell invasion by degrading basement membranes (Atapattu, Lackmann & Janes, 2014), while suppressing EMT markers such as vimentin reduces metastatic potential (Baghbanzadeh et al., 2022). Therefore, the downregulation of these effectors by ESO suggests its ability to interfere with EMT processes, providing a new perspective on how PPIs may impede metastatic dissemination.

In the current study, we also found that ESO reduced the EGF-induced phosphorylation of EGFR, AKT, and mTOR, indicating that inactivation of the EGFR/AKT/mTOR pathway contributes to the inhibitory effect of ESO on EGF-induced invasion and migration in GC cells. The EGFR/AKT/mTOR pathway is a key oncogenic driver in GC and strongly associated with poor prognosis (Cho, 2013; Yang et al., 2025). Upon binding to its ligand, such as EGF, EGFR dimerizes and autophosphorylates, activating several downstream cascades like Ras/Raf/MAPK, AKT/mTOR, and JAK/STAT pathway (Zhang, Wang & Xu, 2023). Among these pathways, the AKT/mTOR pathway is intricately involved in regulating EMT and the expression of MMPs and serves as a central hub that governs the invasive and migratory behavior of cancer cells (Micalizzi et al., 2021; Tang et al., 2017). The central role of AKT signaling in driving tumor progression has been extensively documented across multiple cancer types. For instance, in non-small cell lung cancer, YME1L promotes cancer invasion and migration by enhancing mitochondrial complex I activity *via* its protease function, leading to upregulated Gαi1 expression and subsequent activation of the AKT signaling pathway (Wu et al., 2025). Similarly, in glioblastoma, TRIM29 facilitates cancer invasion and migration by catalyzing K48-linked polyubiquitination and proteasomal degradation of NEFL, thereby relieving its suppression on the PI3K/AKT signaling pathway (Liu et al., 2025).

Afatinib, an irreversible ErbB family blocker, represents a targeted therapy option for EGFR-driven cancers. However, its clinical application in GC has been met with significant challenges, including intrinsic and acquired resistance (Yoshioka et al., 2019). Resistance mechanisms involve the activation of compensatory pathways and EMT (Azuma et al., 2014; Neel & Bivona, 2013). Importantly, our results showed that combining ESO with afatinib led to a significantly stronger inhibition of GC cell migration and invasion compared with monotherapy. Our results are consistent with the previous study that the combination of lansoprazole and gefitinib enhances the inhibition of non-small cell lung cancer (Zhao et al., 2021). The enhanced effect underscores the potential of the combination of ESO and afatinib to improve treatment outcomes in GC.

Our findings provide an experimental basis for exploring the potential value of ESO in the treatment of gastric cancer. However, several limitations of this study should be acknowledged. First, the mechanistic analysis primarily relies on correlative assessments of phosphorylated protein expression within the signaling pathway, without functional causal validation. Future studies should incorporate pathway-specific inhibitors, knockdown or overexpression models of key molecules, along with time-course and rescue experiments to establish the causal role of the EGFR/AKT/mTOR pathway in ESO-mediated inhibition of migration and invasion. Second, our study of the anti-gastric cancer activity of ESO lacks evidence from *in vivo* or clinical studies. Future research should evaluate its *in vivo* antitumor efficacy using animal xenograft models, followed by further validation through retrospective or prospective clinical investigations.

## Conclusions

In conclusion, ESO effectively suppresses EGF-induced invasion and migration in gastric cancer (GC) cells. Notably, the combination of ESO with afatinib performs a synergistic inhibitory effect, markedly enhancing the suppression of malignant phenotypes. Mechanistically, this inhibitory effect is mediated, at least in part, through the inhibition of the EGFR/AKT/mTOR signaling pathway. These findings highlight the potential of ESO as a repurposed anti-tumor agent and support the therapeutic value of combining ESO with EGFR-targeted therapy. Collectively, this strategy may represent a promising approach for the treatment of advanced GC and warrants further investigation in preclinical and clinical settings.

## Additional Information and Declarations

### Data Availability

The following information was supplied regarding data availability:

The data is available at Figshare:

- Huang, Fangfang; Du, Jinfeng; Zhang, Xuanruo; Tian, Mengru; Lv, Qiangqiang; Chu, Xiufeng; et al. (2026). Esomeprazole inhibits EGF-induced invasion and migration of gastric cancer cells *via* suppression of the EGFR/AKT/mTOR pathway. figshare. Dataset. https://doi.org/10.6084/m9.figshare.31026283.v1.
